# In Papyro Comparison of TMM (edgeR), RLE (DESeq2), and MRN Normalization Methods for a Simple Two-Conditions-Without-Replicates RNA-Seq Experimental Design

**DOI:** 10.3389/fgene.2016.00164

**Published:** 2016-09-16

**Authors:** Elie Maza

**Affiliations:** Genomics and Biotechnology of the Fruits Laboratory, UMR 990 INRA/Institut National Polytechnique de Toulouse, Ecole Nationale Supérieure Agronomique de Toulouse, Université de ToulouseCastanet-Tolosan, France

**Keywords:** RNA-seq data, normalization, comparison of methods, DESeq2, edgeR

## Abstract

In the past 5 years, RNA-Seq has become a powerful tool in transcriptome analysis even though computational methods dedicated to the analysis of high-throughput sequencing data are yet to be standardized. It is, however, now commonly accepted that the choice of a normalization procedure is an important step in such a process, for example in differential gene expression analysis. The present article highlights the similarities between three normalization methods: TMM from edgeR R package, RLE from DESeq2 R package, and MRN. Both TMM and DESeq2 are widely used for differential gene expression analysis. This paper introduces properties that show when these three methods will give exactly the same results. These properties are proven mathematically and illustrated by performing *in silico* calculations on a given RNA-Seq data set.

## 1. Introduction

In the past 5 years, RNA-Seq approaches, based on high-throughput sequencing technologies, are becoming an essential tool in transcriptomics studies (cf. Wang et al., 2009). It is now commonly accepted that a normalization preprocessing step can significantly improve the quality of the analysis, in particular, for the differential gene expression analysis (cf. Bullard et al., 2010). Nevertheless, a gold standard normalization method has not yet been found.

This paper deals with two widely used and very important normalization methods and a third method related to these. The first method is the “Trimmed Mean of *M*-values” normalization (TMM) described in Robinson and Oshlack (2010) and implemented in the edgeR package (cf. Robinson et al., 2010). The second method is the “Relative Log Expression” normalization (RLE) implemented in the DESeq2 package (cf. Anders and Huber, 2010; Anders et al., 2013; Love et al., 2014). The third method is the “Median Ratio Normalization” (MRN) described in Maza et al. (2013). It has been shown that TMM and RLE give similar results both with real and simulated data sets (cf. Dillies et al., 2013; Maza et al., 2013; Rapaport et al., 2013; Li et al., 2015; Reddy, 2015). These two methods, as does MRN, deal efficiently with the intrinsic bias resulting from the relative size of studied transcriptomes. Also, it has even been shown that the MRN method performs slightly better on some simulated data sets (cf. Maza et al., 2013). Moreover, many studies have shown that LRE and/or TMM methods outperform other particular methods (cf. Dillies et al., 2013; Maza et al., 2013; Reddy, 2015; Zyprych-Walczak et al., 2015; Lin et al., 2016). Nevertheless, a comprehensive comparison study of differential expression analysis methods has used LRE or TMM for ten of the eleven compared tools (cf. Soneson and Delorenzi, 2013). Finally, other more sophisticated normalization methods have been carried out by iterating one of LRE or TMM methods (cf. Kadota et al., 2012; Sun et al., 2013; Tang et al., 2015).

**Figure 1:**
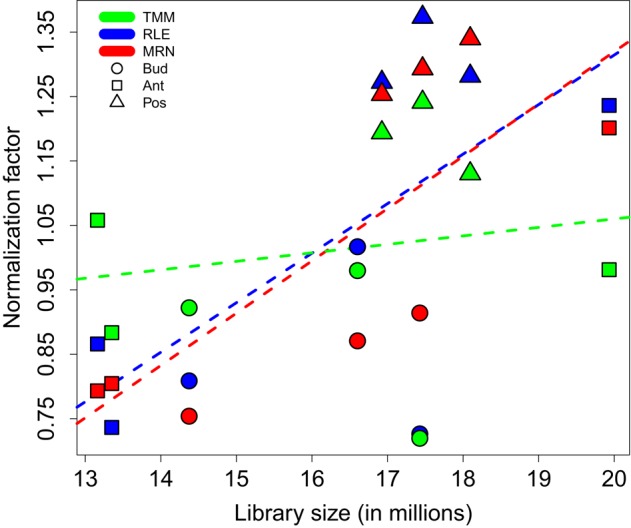
**Normalization factors for the fruit set RNA-Seq data depending on corresponding library sizes**. All three studied normalization methods are carried out with default settings. For all three methods, regression (dashed) lines are estimated from a simple linear regression modeling the relationship between default normalization factors and library sizes. Color key: TMM, RLE, and MRN are respectively colored in green, blue, and red. Key to symbols: Bud, Ant, and Pos stages are respectively drawn with circles, squares, and triangles.

In this paper, all theoretical results will be illustrated by *in silico* calculations carried out on a given real data set from the tomato fruit set (see Materials and Methods). In short, this data set consists of a matrix of counts: 34675 rows (genes) and 9 columns (samples from 3 stages and 3 biological replicates per stage). Normalization factors of these fruit set samples, obtained by each of the TMM, RLE, and MRN methods with default settings, are presented in Table 1. Figure 1 represents the scatter plot of obtained normalization factors and corresponding library sizes. Moreover, Figure 1 contains, for all three normalization methods, the regression lines estimated from a simple linear regression modeling the relationship between default normalization factors and library sizes. It is evident in both Table 1 and Figure 1 that the three methods (with default settings) do not give the same results. Indeed, it is known that TMM normalization factors do not take into account library sizes. This fact is illustrated in Figure 1 by an almost horizontal regression line. On the contrary, RLE and MRN factors are closer to each other, and share a positive correlation with the library size. The estimation of the regression parameters of regression lines above shows that the TMM slope is not statistically significant (at 5% type I error) which is the case of both LRE and MRN slopes (see Additional file 1).

**Table 1:** **Default normalization factors for the fruit set RNA-Seq data**.

**Stage**	**Bud 1**	**Bud 2**	**Bud 3**	**Ant 1**	**Ant 2**	**Ant 3**	**Pos 1**	**Pos 2**	**Pos 3**
TMM	0.98012	0.92236	0.71989	1.05807	0.98130	0.88352	1.13027	1.19388	1.24130
RLE	1.01712	0.80899	0.72660	0.86594	1.23622	0.73647	1.28172	1.27220	1.37315
MRN	0.87105	0.75416	0.91430	0.79324	1.20131	0.80461	1.33984	1.25330	1.29317

*Normalization factors of tomato fruit set samples are obtained from TMM, RLE, and MRN normalization methods with default settings*.

The aim of this study is to provide a deeper understanding as to why the three normalization methods quoted above share a similar normalization approach. This paper also demonstrates that, in some cases, some shared parameters (such as relative size of transcriptomes or normalization factors) are strictly equal.

## 2. Materials and methods

### 2.1. Tomato's RNA-Seq data set

To investigate the tomato transcriptome dynamics of fruit set, RNA were isolated from flower buds (Bud) and flowers at anthesis (Ant) and post-anthesis (Pos) stages. For each stage, cDNA libraries were generated from three biological replicates and subjected to Illumina mRNA-Seq technology sequencing. Then, after mapping reads to the tomato genome sequence, we obtained a table of raw counts with 34675 rows (genes) and 9 columns (3 stages and 3 replicates per stage). These technical procedures are described in Maza et al. (2013). In this paper, for sake of simplicity, the matrix (34675 × 9) containing raw counts is denoted by X.

### 2.2. Computations with R packages

All computations were done within R environment (cf. R Development Core Team, 2011). All packages are available from R or Bioconductor websites (cf. Gentleman et al., 2004).

As described above, the matrix containing raw counts is denoted by X in all R command lines of given *in silico* examples.

The TMM normalization method is implemented in the edgeR package by means of the calcNormFactors function. For example, the default normalization factors obtained in Table 1 are obtained by the following command line:


> calcNormFactors(X)


The RLE normalization method is implemented in the DESeq2 package by means of the function estimateSizeFactorsForMatrix. For example, the default size factors obtained in Table 1 are obtained using the following command line:


> estimateSizeFactorsForMatrix(X)


The MRN normalization method is implemented in a homemade function called mrnFactors which is provided as an additional file (see Additional Files). For example, the default normalization factors obtained in Table 1 are obtained using the following command line:


> mrnFactors(X, rep(1:3 each=3))$normFactors


### 2.3. Definitions of some important terms

We define hereafter some important terms that are used in the studied normalization methods. More detailed definitions are given in Robinson and Oshlack (2010), Anders and Huber (2010), and Maza et al. (2013).

Both *M* and *A*-values are defined for a given gene *g* and its expressions on two conditions *X*_*g*1_ and *X*_*g*2_. They represent respectively the fold change and the absolute expression level of the gene:

$$M=\log_{2}\left(\frac{X_{g1}/N_{1}}{X_{g2}/N_{2}}\right)\text{and}A=\frac{1}{2}\log_{2}\left(\left(X_{g1}/N_{1}\right)\left(X_{g2}/N_{2}\right)\right).$$

A trimmed mean of *M* (respectively *A*)-values corresponds to the calculation of the mean after discarding a given proportion of lower and higher values. A trimmed mean with a proportion of 50% corresponding obviously to the calculation of the median.

## 3. Results and discussion

In this section, the three studied normalization methods are first described and compared. Then, three propositions are introduced and *in silico* results are provided to illustrate them. For the sake of clarity, mathematical proofs of these propositions are left to the end of the section.

We have to underline that we focus, in this paper, on so called “scaling normalization methods” but this is just one approach, which can be limited to some specific experimental situations (cf. Maza et al., 2013). Another alternative can be the use of control genes (see, for instance, Risso et al., 2014).

We notice here that, in order to be consistent, the first paragraph below (named “Notations and Experimental design”) reproduces information that have been already reported in detail in Maza et al. (2013).

### 3.1. Notations and experimental design

Let *X*_*gkr*_ be the observed number of reads (or count) of gene *g* ∈ {1, …, *G*} in condition *k* ∈ {1, …, *K*} for biological replicate *r* ∈ {1, …, *R*}. For the sake of simplicity, we deal with the same number of replicates for each given condition, but the following propositions are not altered by a more general design. Let μ_*gk*_ be the expectation of the true and unknown number of transcripts of a given cell for gene *g* in condition *k*; *L*_*g*_ the length of gene *g*; and *N*_*kr*_ the total number of reads in condition *k* for replicate *r* (or library size). As described in Robinson and Oshlack (2010), we can model the expected value of *X*_*gkr*_ as

$$\begin{array}{l} \text{E}\left(X_{gkr}\right)=\frac{\mu_{gk}L_{g}}{S_{k}}N_{kr} \end{array}$$

where *S*_*k*_ is the size of studied transcriptome in condition *k*, that is

$$\begin{array}{l} S_{k}=\overset{G}{\underset{g=1}{\sum }}\mu_{gk}L_{g}. \end{array}$$

Then, for each gene *g*, an approximation of the expected value of the ratio between two conditions, say 1 and 2, is given by

$$\begin{array}{l} \text{E}\left(\frac{X_{g2r}}{X_{g1r}}\right)\approx \frac{\mu_{g2}}{\mu_{g1}}\frac{S_{1}}{S_{2}}\frac{N_{2r}}{N_{1r}}. \end{array}$$

We can easily see in the equation above that the ratio of interest, in a differential analysis point of view, i.e., $\frac{\mu_{g2}}{\mu_{g1}}$, is not directly measured by ratios of raw counts because of library sizes and relative sizes of transcriptomes. As described in Maza et al. (2013), the three methods studied here aim at removing such biases.

### 3.2. Description of the three methods

Table 2 gives us the description of these three normalization methods. Both TMM and RLE are respectively implemented in edgeR and DESeq2 packages (see Materials and Methods). Differences and commonalities of these three methods are described here, step by step.

**Step I** Both TMM and MRN methods have a step of pre-normalization of counts by library sizes. The RLE method does not and works directly on raw counts.
**Step II** The three methods have a reference sample. For TMM, the reference sample has been chosen here arbitrarily with *k* = 1 and *r* = 1. The default for the edgeR package consists in choosing the library whose upper quartile is closest to the mean upper quartile for all the libraries. For MRN, the condition *k* = 1 is also arbitrarily chosen, as in Maza et al. (2013). Moreover, for MRN, by definition, all replicates of the chosen condition are used. For RLE, a geometric mean of all sample values is performed.
**Step III** For all three methods, relative sizes of transcriptomes and the reference sample are based on ratios of counts (or pre-normalized counts for TMM and MRN) and the reference sample. For TMM, the set $G_{kr}^{*}$ represents the set of not trimmed genes with valid *M* and *A*-values (cf. Robinson and Oshlack, 2010). By default, with the calcNormFactors function of the edgeR package, percentages of trimmed *M* and *A*-values are respectively of 30 and 5% (see Materials and Methods). In order to simplify, but still staying in the same vein, the relative scaling factor calculations are here described with an unweighted trimmed mean instead of the weighted one which is proposed by default on edgeR.
**Step IV** In this step, only the TMM method performs an adjustment of its relative scaling factors to multiply to 1.
**Step V** Only TMM and MRN methods take explicitly into account both relative scaling factors and library sizes.
**Step VI** In this step, it is clear that TMM normalization factors (as produced by the calcNormFactors function) do not take into account the library sizes but only the relative scaling factors. That explains the absence of correlation between normalization factors and library sizes in Figure 1 (see Introduction). In edgeR, these normalization factors are used as offset parameters in the statistical model for differential gene expression analysis. Once again, we underline here that values obtained in Table 1 are not estimations of the same theoretical parameters, and thus, these values can not be used in the same way, for instance, to normalize counts.
**Step VII** For normalization of counts, all three normalization methods take into account both relative sizes of transcriptomes and library sizes. Only the TMM method gives counts-per-million (CPM). Obviously, CPM values can easily be obtained for RLE and MRN methods by multiplying normalized counts by 10^6^.

**Table 2:** **Description of the three normalization methods**.

**Step**	**Description**	**TMM (edgeR)**	**RLE (DESeq2)**	**MRN**
I	Pre-normalization by library size	$Y_{gkr}=\frac{X_{gkr}}{N_{kr}}$		$Y_{gkr}=\frac{X_{gkr}}{N_{kr}}$
II	Reference sample, or *pseudo-reference sample* (DESeq2)	$Y_{g}^{\text{TMM}}=Yg11$	$Y_{g}^{\text{RLE}}=\sqrt[{KR}]{\prod_{k\;=\;1}^{K}\prod_{r\;=\;1}^{R}X_{gkr}}$	$Y_{g}^{\text{MRN}}=\frac{1}{R}\sum_{r\;=\;1}^{R}Y_{g1r}$
III	Relative sizes of transcriptomes and reference sample, or *relative scaling factors* (edgeR), or *size factors* (DESeq2)	$\tau_{kr}^{\text{TMM}}=\frac{1}{\#G_{kr}^{*}}\sum_{g\in G_{kr}^{*}}\frac{Y_{gkr}}{Y_{g}^{\text{TMM}}}$ *where* $G_{kr}^{*}$ represents the set of not trimmed genes	$\tau_{kr}^{\text{RLE}}=\underset{g}{\text{median}}\left(\frac{X_{gkr}}{Y_{g}^{\text{LRE}}}\right)$	$\tau_{k}^{\text{MRN}}=\underset{g}{\text{median}}\left(\frac{Ȳgk}{Y_{g}^{\text{MRN}}}\right)$ where ${\bar{Y}}_{gk}=\frac{1}{R}\sum_{r=1}^{R}Y_{gkr}$
IV	*Relative scaling factors* adjusted to multiply to 1 (edgeR)	$\begin{array}{l} {\tilde{\tau }}_{kr}^{\text{TMM}}=\frac{\tau_{kr}^{\text{TMM}}}{{\tilde{\tau }}^{\text{TMM}}}\text{ where}\\ {\tilde{\tau }}^{\text{TMM}}=\sqrt[{KR}]{\prod_{k\;=\;1}^{K}\prod_{r\;=\;1}^{R}\tau_{kr}^{\text{TMM}}} \end{array}$		
V	Taking into account both the relative size and the library size, or *effective library size* (edgeR)	$e_{kr}^{\text{TMM}}={\tilde{\tau }}_{kr}^{\text{TMM}}N_{kr}$		$e_{kr}^{\text{MRN}}=\tau_{k}^{\text{MRN}}N_{kr}$
VI	Normalization factors, or *relative normalization factors* (edgeR), or *size factors* (DESeq2)	$f_{kr}^{\text{TMM}}={\tilde{\tau }}_{kr}^{\text{TMM}}$	$f_{kr}^{\text{RLE}}=\tau_{kr}^{\text{RLE}}$	$\begin{array}{l} f_{kr}^{\text{MRN}}=\frac{e_{kr}^{\text{MRN}}}{{\tilde{e}}^{\text{MRN}}}\text{ where}\\ {\tilde{e}}^{\text{MRN}}=\sqrt[{KR}]{\prod_{k\;=\;1}^{K}\prod_{r\;=\;1}^{R}e_{kr}^{\text{MRN}}} \end{array}$
VII	Normalization of counts, or *counts-per-million* (edgeR)	$Z_{gkr}^{\text{TMM}}=\frac{X_{gkr}}{e_{kr}^{\text{TMM}}}10^{6}$	$Z_{gkr}^{\text{RLE}}=\frac{X_{gkr}}{f_{kr}^{\text{RLE}}}$	$Z_{gkr}^{\text{MRN}}=\frac{X_{gkr}}{f_{kr}^{\text{MRN}}}$

### 3.3. Properties of the three methods

After the above detailed descriptions of our three methods, we introduce below three properties showing particular cases where all three methods give the same result.

Proposition 1(concerning TMM and MRN). *Let's assume that the following assumptions hold for the calculus of the TMM normalization method:*

- *The reference sample is (arbitrarily) the first one (k* = 1 *and r* = 1).
- *Trimming parameters of M and A-values are respectively equal to 50 and 0%*.
- *Calculation of the trimmed mean is done without computing weights (unweighted mean)*.

*Moreover, let R* = 1 *(no replicates). Then, the relative scaling factors of TMM and MRN methods are equal:*

$$\tau_{k1}^{TMM}=\tau_{k}^{MRN}.$$

An example illustrating Proposition 1 is given in Table 3. Calculations are carried out by means of R functions calNormFactors (from the edgeR package) for the TMM method and mrnFactors (see Materials and Methods) for the MRN method, as follows:


> calcNormFactors(X, refColumn=1,
logratioTrim=0.499, sumTrim=0,
doWeighting=FALSE)
> mrnFactors(X, 1:9)$medianRatios


**Table 3:** **Normalization factors of tomato fruit set samples, obtained from TMM and MRN normalization methods with parameters of Proposition 1**.

**Stage**	**Bud 1**	**Bud 2**	**Bud 3**	**Ant 1**	**Ant 2**	**Ant 3**	**Pos 1**	**Pos 2**	**Pos 3**
TMM	0.97654	0.92966	0.72054	1.06259	0.97360	0.87363	1.14166	1.19541	1.23937
MRN	0.97658	0.92957	0.72079	1.06280	0.97361	0.87361	1.14189	1.19599	1.23792

We can clearly see in Table 3 that, with function arguments corresponding to the assumptions of Proposition 1, the adjusted relative scaling factors produced by TMM and MRN methods are equal up to the third or fourth decimal place for almost all of the values (only one of the values has just two decimal places equal). This slight difference is due to the logratioTrim argument that cannot be strictly equal to 50%.

Proposition 2 (concerning RLE and MRN). *Let K* = 2 *conditions and no replicates (R* = 1*). Then, the size factors calculated from the RLE and MRN methods are equal:*

$$f_{k1}^{RLE}=f_{k1}^{MRN}.$$

We illustrate Proposition 2 by calculating the size factors for some pairs of samples (see Table 4). Calculations are carried out by means of R functions estimateSizeFactorsForMatrix (from the DESeq2 package) for the RLE method and mrnFactors (see Materials and Methods) for the MRN method, as follows:


> estimateSizeFactorsForMatrix(X[ , c(1,
2)])
> mrnFactors(X[ , c(1, 2)], c(1,
2))$normFactors
> estimateSizeFactorsForMatrix(X[ , c(3,
5)])
> mrnFactors(X[ , c(3, 5)], c(1,
2))$normFactors
> estimateSizeFactorsForMatrix(X[ , c(4,
7)])
> mrnFactors(X[ , c(4, 7)], c(1,
2))$normFactors


**Table 4:** **Normalization factors of some pairs of tomato fruit set samples, obtained from RLE and MRN normalization methods with parameters of Proposition 2**.

**Stage**	**Bud 1**	**Bud 2**	**Bud 3**	**Ant 2**	**Ant 1**	**Pos 1**
RLE	1.1015522	0.9078099	0.7870385	1.2705859	0.8248517	1.2123391
MRN	1.1015522	0.9078099	0.7870385	1.2705859	0.8248517	1.2123391

We can see in Table 4 that, as introduced in Proposition 2, normalization results from both methods are equal.

We must note here that, with *K* > 2, Proposition 2 does not hold: with more than two samples, the reference sample of the RLE method takes into account all raw counts and this is obviously not the case for the MRN method. This can be checked by straightforward calculations with more than two samples.

Proposition 3 (concerning RLE, TMM, and MRN). *Let's assume that the assumptions of both Proposition 1 and Proposition 2 are satisfied. Then, normalized counts of the RLE and MRN methods, and counts-per-million of the TMM method (up to a constant) are equal:*

$$Z_{gk1}^{TMM}\times \frac{\sqrt{N_{11}N_{21}}}{10^{6}}=Z_{gk1}^{RLE}=Z_{gk1}^{MRN}.$$

### 3.4. Calculation of RLE normalization factors with edgeR

We note here that the calcNormFactors function contains an argument called “method” that can also be used to calculate RLE normalization factors (cf. the description of the calcNormFactors function in the edgeR package). The user should however be careful! Indeed, as calculated below, calcNormFactors with method="RLE" and estimateSizeFactorsForMatrix functions do not give the same results. What happens is that the calcNormFactors function does not work with raw counts but with pre-normalized ones (see Step I of Table 2). Moreover, the calcNormFactors function proceeds to an adjustment of values (see Step IV of Table 2). In order to find the same values with both functions, we have to proceed as follows:


> calcNormFactors(X, method="RLE")
> estimateSizeFactorsForMatrix(X)
> f=estimateSizeFactorsForMatrix(X%*%diag
(1/colSums(X)))
> f/prod(f) ^(1/length(f))


### 3.5. Proofs of propositions

*Proof of Proposition 1*. We first note that, with *R* = 1, i.e., with no biological replicates, the reference samples of both TMM and MRN methods are equal:

$$Y_{g}^{\text{TMM}}=Y_{g}^{\text{MRN}}.$$

Moreover, if we assume that (i) the trimmed mean of the TMM method is done with an unweighted mean as described in the Step III of the edgeR method in Table 2, and that (ii) the trimming values are equal to 50% of genes with upper *M*-values and 50% of genes with lower *M*-values, then we obtain that

$$\tau_{k1}^{\text{TMM}}=\frac{1}{\#G_{k1}^{*}}\underset{g\in G_{k1}^{*}}{\sum }\frac{Y_{gk1}}{Y_{g}^{\text{TMM}}}=\underset{g}{\text{median}}\left(\frac{Y_{gk1}}{Y_{g}^{\text{TMM}}}\right).$$

Then

$$\tau_{k1}^{\text{TMM}}=\underset{g}{\text{median}}\left(\frac{Y_{gk1}}{Y_{g}^{\text{MRN}}}\right)=\tau_{k}^{\text{MRN}}.$$

                         □

*Proof of Proposition 2*. Let's first describe the RLE method calculations by following steps of Table 2. For *K* = 2 and *R* = 1, the pseudo-reference sample is the following:

$$Y_{g}^{\text{RLE}}=\sqrt{X_{g11}X_{g21}}.$$

Then, we directly have that

$$f_{11}^{\text{RLE}}=\tau_{11}^{\text{RLE}}=\underset{g}{\text{median}}\left(\sqrt{\frac{X_{g11}}{X_{g21}}}\right)$$

and

$$f_{21}^{\text{RLE}}=\tau_{21}^{\text{RLE}}=\underset{g}{\text{median}}\left(\sqrt{\frac{X_{g21}}{X_{g11}}}\right).$$

Let's then describe calculations for the MRN method. For *K* = 2 and *R* = 1, the reference sample is simply the first sample:

$$Y_{g}^{\text{MRN}}=Y_{g11}.$$

Then, the relative sizes are the following:

$$\tau_{1}^{\text{MRN}}=1\text{and}\tau_{2}^{\text{MRN}}=\underset{g}{\text{median}}\left(\frac{Y_{g21}}{Y_{g11}}\right)=\underset{g}{\text{median}}\left(\frac{X_{g21}}{X_{g11}}\right)\frac{N_{11}}{N_{21}}.$$

That leads to

$$e_{11}^{\text{MRN}}=N_{11}\text{and}e_{21}^{\text{MRN}}=\underset{g}{\text{median}}\left(\frac{X_{g21}}{X_{g11}}\right)N_{11}.$$

Finally, the calculation of the geometric mean of these values, i.e.,

$${ẽ}^{\text{MRN}}=\underset{g}{\text{median}}\left(\sqrt{\frac{X_{g21}}{X_{g11}}}\right)N_{11}$$

implies that

$$f_{11}^{\text{MRN}}=\underset{g}{\text{median}}\left(\sqrt{\frac{X_{g11}}{X_{g21}}}\right)$$

and

$$f_{21}^{\text{MRN}}=\underset{g}{\text{median}}\left(\sqrt{\frac{X_{g21}}{X_{g11}}}\right).$$

It follows that

$$f_{k1}^{\text{MRN}}=f_{k1}^{\text{RLE}}.$$

                         □

*Proof of Proposition 3*. We have already proven that, assuming the assumptions of Proposition 2, i.e., *K* = 2 and *R* = 1, the RLE and MRN methods produce the same normalization factors. Then, obviously, normalized counts are equal. Let's then prove that TMM and MRN normalized counts are equal up to a constant.

For the TMM method, assuming the assumptions of Proposition 1, the relative scaling factors are the following:

$$\tau_{11}^{\text{TMM}}=\tau_{1}^{\text{MRN}}=1$$

and

$$\tau_{21}^{\text{TMM}}=\tau_{2}^{\text{MRN}}=\underset{g}{\text{median}}\left(\frac{X_{g21}}{X_{g11}}\right)\frac{N_{11}}{N_{21}}.$$

Then, with the following geometric mean of these values:

$${\tilde{\tau }}^{\text{TMM}}=\sqrt{\tau_{11}^{\text{TMM}}\tau_{21}^{\text{TMM}}}=\underset{g}{\text{median}}\left(\sqrt{\frac{X_{g21}}{X_{g11}}}\right)\sqrt{\frac{N_{11}}{N_{21}}}$$

the adjusted relative scaling factors are the following:

$${\tilde{\tau }}_{11}^{\text{TMM}}=\underset{g}{\text{median}}\left(\sqrt{\frac{X_{g11}}{X_{g21}}}\right)\sqrt{\frac{N_{21}}{N_{11}}}$$

and

$${\tilde{\tau }}_{21}^{\text{TMM}}=\underset{g}{\text{median}}\left(\sqrt{\frac{X_{g21}}{X_{g11}}}\right)\sqrt{\frac{N_{11}}{N_{21}}}.$$

We can then calculate the effective library sizes:

$$e_{11}^{\text{TMM}}={\tilde{\tau }}_{11}^{\text{TMM}}N_{11}=\underset{g}{\text{median}}\left(\sqrt{\frac{X_{g11}}{X_{g21}}}\right)\sqrt{N_{11}N_{21}}$$

and

$$e_{21}^{\text{TMM}}={\tilde{\tau }}_{21}^{\text{TMM}}N_{21}=\underset{g}{\text{median}}\left(\sqrt{\frac{X_{g21}}{X_{g11}}}\right)\sqrt{N_{11}N_{21}}.$$

Hence, these effective library sizes are equal (up to a constant) to the normalization factors obtained from RLE (and MRN) methods:

$$e_{11}^{\text{TMM}}=f_{11}^{\text{RLE}}\sqrt{N_{11}N_{21}}$$

and

$$e_{21}^{\text{TMM}}=f_{21}^{\text{RLE}}\sqrt{N_{11}N_{21}}.$$

And the proposition is proved.     □

## 4. Conclusions and further work

This paper focus on two widely used normalization methods for RNA-Seq data and a third method related to these, that seem to give similar results and outperform many other classical methods if we consider all references given in the Introduction. Better understanding these methods is then an important issue dealt by this paper.

We highlight in this paper that the three considered normalization methods deal with similar underlying ideas. Moreover, we prove that these methods give exactly the same result in some simple experimental designs. For instance, Proposition 3 shows that for two given samples, normalized counts are (up to a constant) equal.

It has also been shown in this paper that the user should carefully use and not mix these normalization methods and R packages as all concepts are not equal. For instance, the so called “normalization factors” from edgeR and “size factors” from DESeq2 are not the same theoretical parameters.

Nevertheless, it has been shown in Maza et al. (2013) that the MRN method performs slightly better on some simulated data sets with a standard experimental design of two conditions with replicates. The present paper does not explain why it performs better but attempts to give some hypotheses, inspired by the proved propositions, by focusing on what differ between the three normalization methods. We give hereafter some of these hypotheses. (i) For instance, the reference sample of the MRN method, as a mean of all replicates of a given condition, is a more robust estimation of mean counts in a given condition (more robust than the TMM method). (ii) Also, for the TMM method, the trimming parameter of *M*-values should perhaps (by default) be chosen around 50% in order to have a more robust estimation of the relative size of transcriptomes. (iii) Moreover, in the same way, for the MRN method, the relative sizes of transcriptomes are not sample-specific but condition-specific. Indeed, for the MRN method, these relative sizes are the same for all replicates of a condition. This should perhaps give a more robust estimation than for TMM and RLE relative sizes. All these hypotheses, among others, should be explored in forthcoming work.

Finally, we conclude here that for a very simple experimental design, i.e., about two conditions and no replicates, users can use any of the three studied normalization methods with no impact on results. But, for a more complex experimental design, the results described in Maza et al. (2013) tend to indicate that the MRN method could be adopted. However, obviously, this last hypothesis should be proved rigorously in further work.

## Author contributions

EM has carried out the calculations, performed the analysis, and written the paper.

### Conflict of interest statement

The author declares that the research was conducted in the absence of any commercial or financial relationships that could be construed as a potential conflict of interest. The reviewer CK and handling Editor declared their shared affiliation, and the handling Editor states that the process nevertheless met the standards of a fair and objective review.

## Additional files

### Additional file 1 — script of R commands

This first additional file contains the R code of all calculations carried out in this paper. This file can be obviously executed on R directly (see Materials and Methods) but it can also be opened with a simple text viewer.

### Additional file 2 — script of mrnFactors function

The second additional file contains the R code for the mrnFactors function (see Materials and Methods). As the additional file above, this file can be executed on R directly and can be opened with a simple text viewer. This file is called by Additional file 1.

### Additional file 3 — fruit set data

This file contains the fruit set data used in Additional file 1. This file is a simple text file.
